# “Nutrient–fungi–host” tripartite interaction in cancer progression

**DOI:** 10.1002/imt2.170

**Published:** 2024-01-26

**Authors:** Di Wu, Yun‐Xuan Guan, Chen‐Hao Li, Quan Zheng, Zuo‐Jing Yin, Hui Wang, Ning‐Ning Liu

**Affiliations:** ^1^ State Key Laboratory of Systems Medicine for Cancer, Center for Single‐Cell Omics, School of Public Health Shanghai Jiao Tong University School of Medicine Shanghai China; ^2^ Institute of Computing Technology Chinese Academy of Sciences Beijing China

**Keywords:** anticancer treatment, cancer, fungi, mycobiome, nutrients

## Abstract

The human microbiome exhibits a profound connection with the cancer development, progression, and therapeutic response, with particular emphasis on its components of the mycobiome, which are still in the early stages of research. In this review, we comprehensively summarize cancer‐related symbiotic and pathogenic fungal genera. The intricate mechanisms through which fungi impact cancer as an integral member of both gut and tissue‐resident microbiomes are further discussed. In addition, we shed light on the pivotal physiological roles of various nutrients, including cholesterol, carbohydrates, proteins and minerals, in facilitating the growth, reproduction, and invasive pathogenesis of the fungi. While our exploration of the interplay between nutrients and cancer, mediated by the mycobiome, is ongoing, the current findings have yet to yield conclusive results. Thus, delving into the relationship between nutrients and fungal pathogenesis in cancer development and progression would provide valuable insights into anticancer therapy and foster precision nutrition and individualized treatments that target fungi from bench to bedside.

## Highlights

- Symbiotic and pathogenic fungi are crucial in modulating various processes of cancer.
- Dietary nutrients play essential physiological roles in growth, reproduction, and invasive pathogenesis of fungi.
- The interactions between nutrients and fungi in cancer development provide valuable insights into the application of fungi‐targeted strategies in precision nutrition for cancer prevention and treatment.

## Graphical abstract

Interactions between nutrient and fungi in cancer development and treatment remain under investigation. In this article, we aim to provide insights into the relationships between fungi and various categories of cancer, mediated by diverse nutrient sensing pathways of fungi in response to external environment. Moreover, we highlight the potential application of fungi‐targeted strategies in precision nutrition for both cancer prevention and treatment.

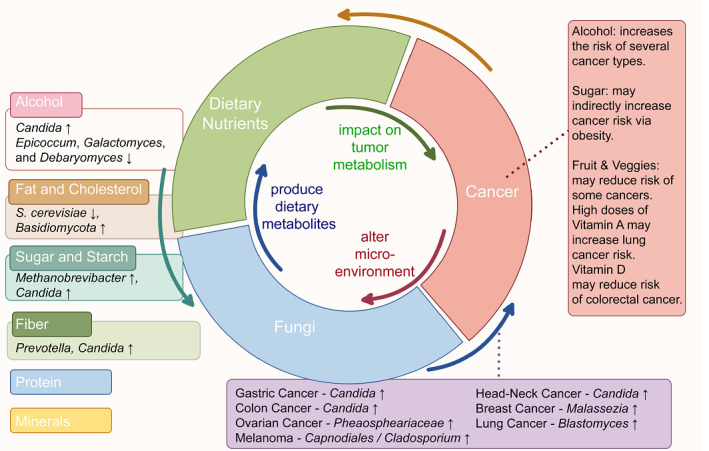

## INTRODUCTION

Cancer, as a leading cause of global mortality, was responsible for approximately 10 million deaths in 2020 [1, 2]. The microbiota, a major component of the human body, is indispensable for maintaining overall health. The role of bacteria in cancer has received growing attention over the past decades. The importance of fungi, another essential taxon of the microbiota, has often been overlooked. Although much larger in size than bacteria, fungi account for only 0.1% of microbial DNA, leading to their underestimation as potential disease‐causing agents [3]. Fungal infections contribute to over 1.5 million fatalities worldwide annually and have a significant impact on the host immune system and microbiota as integral components [4, 5].

Recent studies have shed light on the presence of fungal mycobiome across various cancer types, signaling a shift in cancer microbiome research from the bacteriome to the mycobiome, marking a new era in mycobiome‐driven cancer research [6, 7, 8, 9]. Intriguingly, several studies have moved beyond the compositional correlation between fungi and cancer and have started unraveling the intricate mechanisms driving fungal involvement in pancreatic and lung cancer, thereby transitioning from association to causation in cancer mycobiome research [10, 11, 12]. However, these mechanistic studies are still limited to association between specific fungi and tumor types, which awaits the further investigation of molecular mechanisms underlying fungal‐driven tumorigenesis and targeted therapeutic pathways.

**Figure 1:**
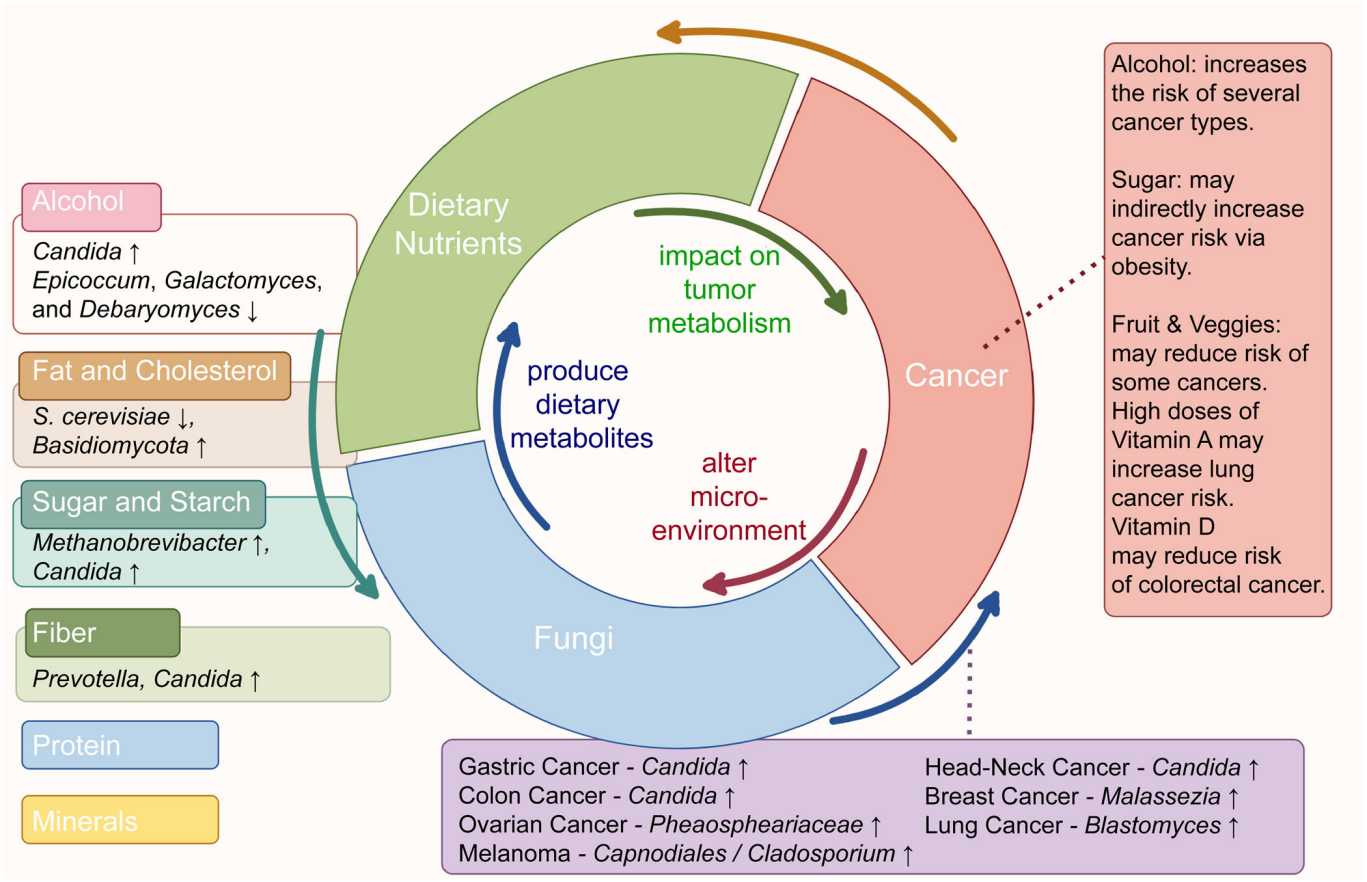
“Nutrition–fungi–host” tripartite interaction in cancer progression. Mycobiome represents a putative correlation between nutrients and cancer. Both commensal and pathogenic fungi are involved in regulation of oncogenesis progress, through secreting bioactive molecules, modulating host immunity, and cross‐talking with nearby bacteria. ↓, decrease; ↑, increase.

Current research on fungi and tumors has made significant strides, revealing a need for extensive supplementation, particularly in the investigation of tumor‐associated microbiota. Nutrients have been shown to play a crucial role in fungal growth, proliferation, invasion, pathogenesis, as well as the composition and diversity of the mycobiota [13, 14]. For instance, glucose could induce the morphological transition of *Candida albicans* from yeast to hyphae, enhancing its ability to adapt to environmental stress, thereby facilitating fungal colonization and host invasion [15, 16]. Hence, tumor progression and nutrition are intimately correlated. Adequate nutritional intake reduces susceptibility to tumor development, while excessive intake may have the opposite effect. For example, sufficient folate and calcium intake can lower the risk of colon carcinogenesis, whereas high doses of β‐carotene may increase the risk of lung cancer [17]. However, establishing a causal relationship between nutrients and tumors is challenging, and our understanding of the pathogenic mechanisms by which fungi are regulated by nutrients is still limited. Furthermore, there is a growing interest in exploring the role of mycobiota and precision nutrition in cancer prevention and treatment. In this review, we aim to provide an overview of the connection between mycobiota and specific tumor types, as well as the differential nutrient sensing exhibited by fungi in response to the external environment. We also highlight the application of fungi‐targeted strategies in precision nutrition and cancer prevention (Figure 1).

## FUNGI AND CANCER

**Figure 2:**
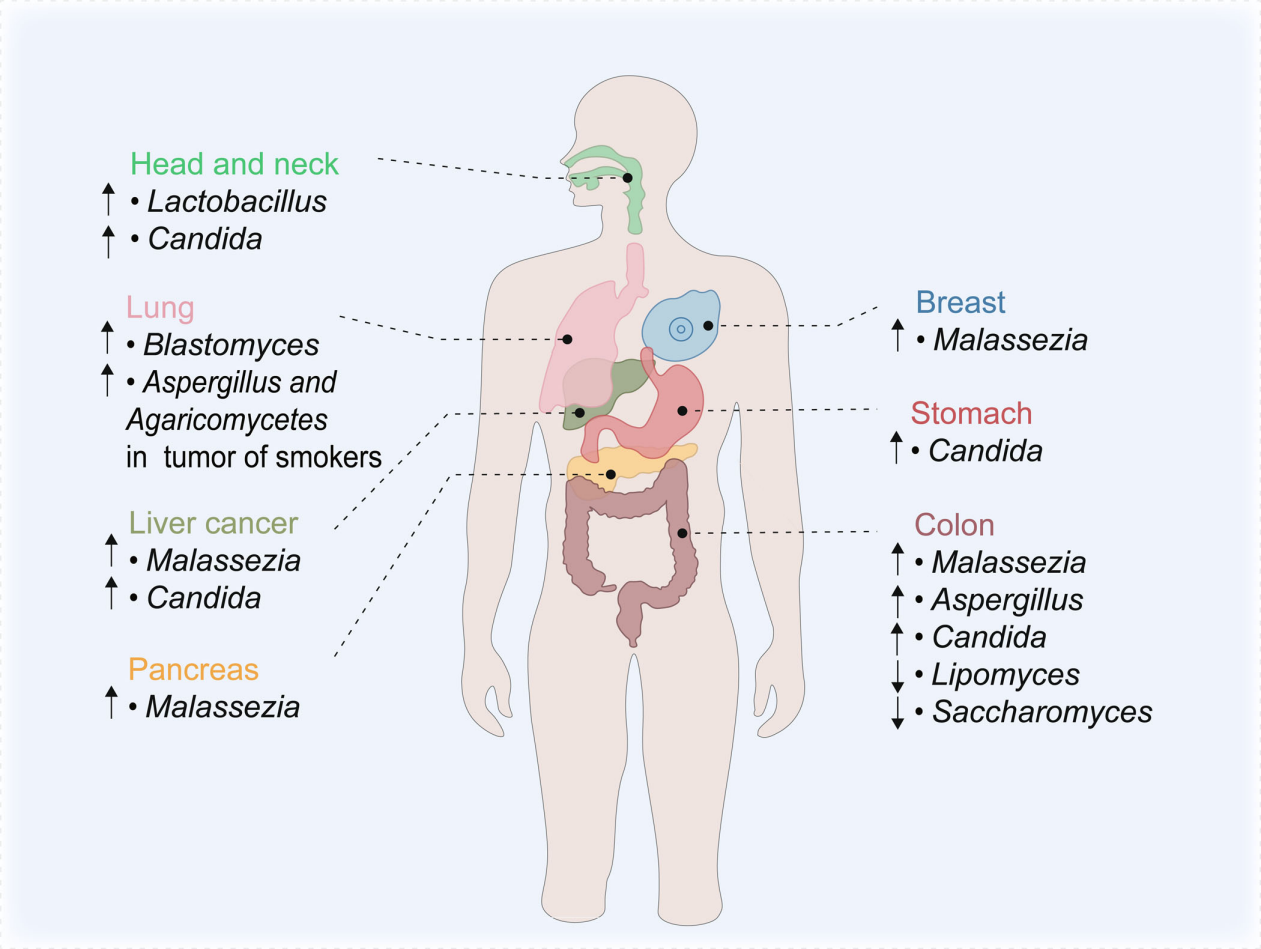
The alteration of mycobiome in abundance across different tumor sites. The composition of fungal mycobiome found to be altered in different body sites (colorectum, pancreas, stomach, liver, head and neck, lung, and breast) that are associated with tumourigenesis, and may serve as potential diagnostic or prognostic biomarkers to promote the study of the complicated mechanistic investigation of fungal involvement in carcinogenesis. ↓, decrease; ↑, increase.

The abundance of various bacterial species in the mucosal microbiota and within the tumors has been extensively studied in relation to different types of cancers over the past decades. However, emerging research has highlighted the significance of fungal strains in the context of cancer. Similar to bacteria, the composition of fungal strains shows variations depending on the tumor site, and the presence of cancer‐specific fungi provides new ideas for tumor‐related detection and diagnosis. These correlations in fungal composition offer a promising starting point for exploring the involvement of fungal commensal in carcinogenesis through intricate mechanisms, which may open up avenues for innovative therapeutic strategies and improved patient outcomes. In this section, we delve into the relationship between fungi and cancer, highlighting key findings and exploring potential mechanisms (Figure 2).

### Digestive system cancers

#### Colorectal cancer (CRC)

Globally, CRC is one of the most common causes of human deaths, and the incidence is rising, with approximately 900,000 deaths each year [18, 19, 20]. Risk factors such as diet, lifestyle, and environment may alter the composition and ecological properties of gut microbiota, which may be involved in the metabolic and immune pathways in colorectal tumorigenesis [21].

Indeed, the role of fungi in CRC is an emerging area of research, and several studies have investigated the alterations in the composition and function of mycobiota in CRC patients [3, 20, 22]. The findings suggest that fungal dysbiosis and changes in the fungal network are critical to the pathogenesis of CRC.

Studies using shotgun metagenomics have revealed specific alterations in the fungal composition in CRC patients compared with healthy controls. These alterations include an enrichment of *Basidiomycota*/*Ascomycota* ratio [23], an abundance of *Malasseziomycetes*, and a depletion of *Saccharomycetes* and *Pneumocystidomycetes*. Furthermore, specific fungal species such as *Lipomyces starkeyi* and *Saccharomyces cerevisiae* were found to be reduced, while others like *Malassezia globosa* and *Aspergillus flavus* were enriched in CRC patients [3].

The characteristic fungal abundance in CRC patients has shown promise as a potential biomarker for CRC diagnosis. Fecal fungal signatures have been identified with high diagnostic accuracy, distinguishing CRC patients from healthy subjects. Multikingdom models that incorporate fungal species have demonstrated improved diagnostic performance compared with single‐kingdom models, emphasizing the value of fungi as biomarkers for CRC diagnosis [20, 22].

Moreover, colon tumors were shown to contain more fungal signals compared with negative controls in the studies of intratumoral mycobiota, with the highest *Ascomycota* to *Basidiomycota* ratio of which *C. albicans* being the most prevalent fungal species [6, 8]. In addition, there were differences in the relative abundance of *C. albicans* and *S. cerevisiae* in gastrointestinal tumors, indicating that they might be classified into *Candida‐* and *Saccharomyces*‐associated tumors. Intriguingly, transcriptional downregulation of genes associated with cell and focal adhesion, extracellular matrix receptor interactions, and tumor suppressors was discovered in colon tumors containing *C. albicans*, which were also inclined to metastasize [6].

**Figure 3:**
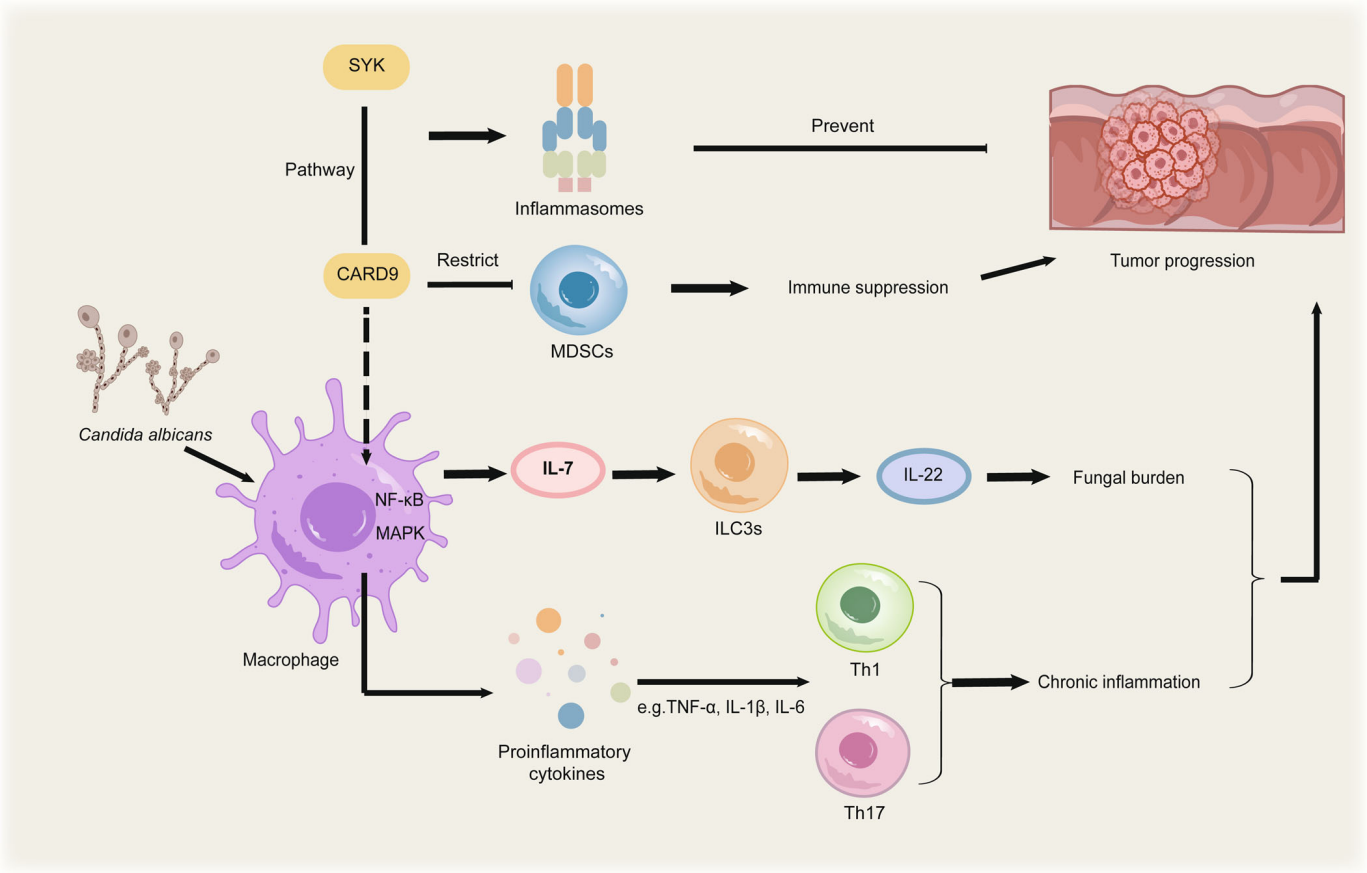
Illustration of the different mechanisms by which fungi promote the development of colorectal cancer (CRC). Increased abundance of *Candida albicans* can upregulate the production of IL‐7 and IL‐22 in macrophages and RORct^+^ ILC3s, respectively. The level of IL‐22 and MDSCs was strongly associated with increased fungal burden. The adapter protein CARD9 could restrict MDSCs and activate the NF‐κB and MAPK pathways in macrophages, leading to the production of proinflammatory cytokine cascades and activation of Th1 and Th17 cells subsequently. Depletion of *Card9* in macrophages can result in elevated fungal loads, which trigger MDSCs differentiation and maturation. Furthermore, the SYK‐CARD9 signaling axis could activate fungal‐mediated inflammasomes, thereby preventing CRC progression. CARD9, caspase‐recruitment domain 9; IL‐7, interleukin‐7; ILC3s, group 3 innate lymphoid cells; MAPK, mitogen‐activated protein kinase; MDSCs, myeloid‐derived suppressor cells; NF‐κB, nuclear factor kappa B; SYK, spleen tyrosine kinase; TNF‐α, tumour necrosis factor‐alpha.

Recently, some studies have also found definitive evidence that fungi are responsible for tumorigenesis of CRC. Increased *C. albicans* in CRC samples can regulate interleukin‐7 (IL‐7) production through HIF‐1‐dependent glycolysis in macrophages, leading to the production of IL‐22 in RORct^+^ group 3 innate lymphoid cells controlled by transcriptional factor Stat3 and AhR. Particularly, the level of IL‐22 in the tumor tissues was strongly linked with fungal burden [24]. The frequency of myeloid‐derived suppressor cells (MDSCs) in colon cancer tissues has been significantly correlated with the fungal loads, demonstrating the importance of the regulatory axis in human disease [25]. While MDSCs could enhance immune suppression and promote tumor growth [26], adapter protein caspase‐recruitment domain 9 (CARD9) could restrict MDSCs thus preventing colon cancer. Moreover, CARD9 is a signaling protein that activates the nuclear factor kappa B and mitogen‐activated protein kinase (MAPK) pathways in macrophages, leading to the production of proinflammatory cytokine cascades [27]. Consequentially, the proinflammatory cytokines such as tumour necrosis factor‐alpha, IL‐1β, and IL‐6 could activate Th1 and Th17 cells, which stimulated phagocyte activation and neutrophil recruitment and in turn lead to chronic inflammation [28]. Additionally, *Card9*‐deficient macrophages with lower fungicidal activity resulted in elevated fungal loads and variance in the overall makeup of the gut mycobiota, considerably rising in *Candida tropicalis*, which triggered MDSCs differentiation and activated the function [25]. The SYK‐CARD9 signaling axis could activate fungal‐mediated inflammasomes thereby preventing CRC. On the contrary, the deletion of CARD9 or SYK in myeloid cells inhibited IL‐18 maturation and inflammasome activation, and raised susceptibility to CRC by elevating fungi [29]. Generally, these discoveries advance our knowledge of how commensal mycobiota control host immunity and facilitate carcinogenesis by establishing *Candida*‐driven interactions (Figure 3).

Besides, pathogenic fungi could secrete detrimental metabolites such as acetaldehyde from *C. albicans* and *C. tropicalis*, and candidalysin from *C. albicans*, which disrupt the epithelial barrier function and induce cytotoxicity and DNA damage, exerting different levels of carcinogenic effects [30]. Interaction between bacteria and mycobiota is equally, if not more, important in the development of CRC carcinogenesis, which can result in the formation of pathogenic biofilms. *C. albicans* and bacterial microorganisms including *Escherichia coli*, *Enterococcus faecalis*, *Streptococcus* spp., *Staphylococcus aureus*, and *Staphylococcus epidermidis* synergistically promote the formation of biofilms, which protect microorganisms from the host immune system, increase epithelial permeability, and promote local inflammatory responses [31, 32]. Hence, colonic mucosal biofilms may be triggers of CRC and have the potential to create a perfect environment of persistent inflammation for the promotion of colon carcinogenesis [33].

Last but not least, a growing body of evidence suggests that there are important interactions between intestinal stem cells and gut microbiota, which can influence their proliferation, differentiation, reprogramming and transformation into tumor stem cells, finally leading to the progression of CRC. However, these studies have primarily focused on the role of bacteria in abnormal reprogramming of tumor and intestinal stem cells, whereas the involvement of fungi has not been fully explored. Further research is needed to thoroughly reveal the significance of fungi in the development of CRC at the intestinal and tumor stem cell levels.

Overall, the studies conducted thus far have provided valuable insights into the association between mycobiota and CRC, highlighting the potential of fungi as diagnostic biomarkers and their involvement in tumorigenesis and immune responses. Further research in this area will deepen our understanding of the complex interactions between fungi, the gut microbiota, and CRC pathogenesis.

#### Pancreatic cancer

The global burden of pancreatic cancer has increased sharply over the past few decades and is predicted to remain the main cause of cancer‐related deaths [34]. Nonetheless, the precise contribution of mycobiota to the development of pancreatic ductal adenocarcinoma (PDA) has remained elusive. In a cohort from the United States, Aykut et al. found that fungi in PDA tumors were ~3000‐fold abundant compared with normal pancreas in both mice and human studies, and differed obviously from the gut or normal pancreas in α and β diversity, with significant enrichment of *Malassezia* in particular [11]. Fungal ablation prevents tumors in a slowly progressing model of invasive PDA, whereas repopulation with *Malassezia* instead of other commensal fungi speeds up tumorigenesis by activating ligation of mannose‐binding lectin (MBL) to drive the complement cascade. MBL, a lectin that recognizes fungal infections and activates the pathway of the complement cascade, is essential for carcinogenesis development [35], while C3aR knockdown in tumor cells or deletion of MBL or C3 in the extra‐tumoral compartment are protective against tumor growth. Meanwhile, oncogenic *Kras*‐induced inflammation results in fungal dysbiosis, which subsequently activates the MBL‐C3 cascade to promote the proliferation of tumors [11].

**Figure 4:**
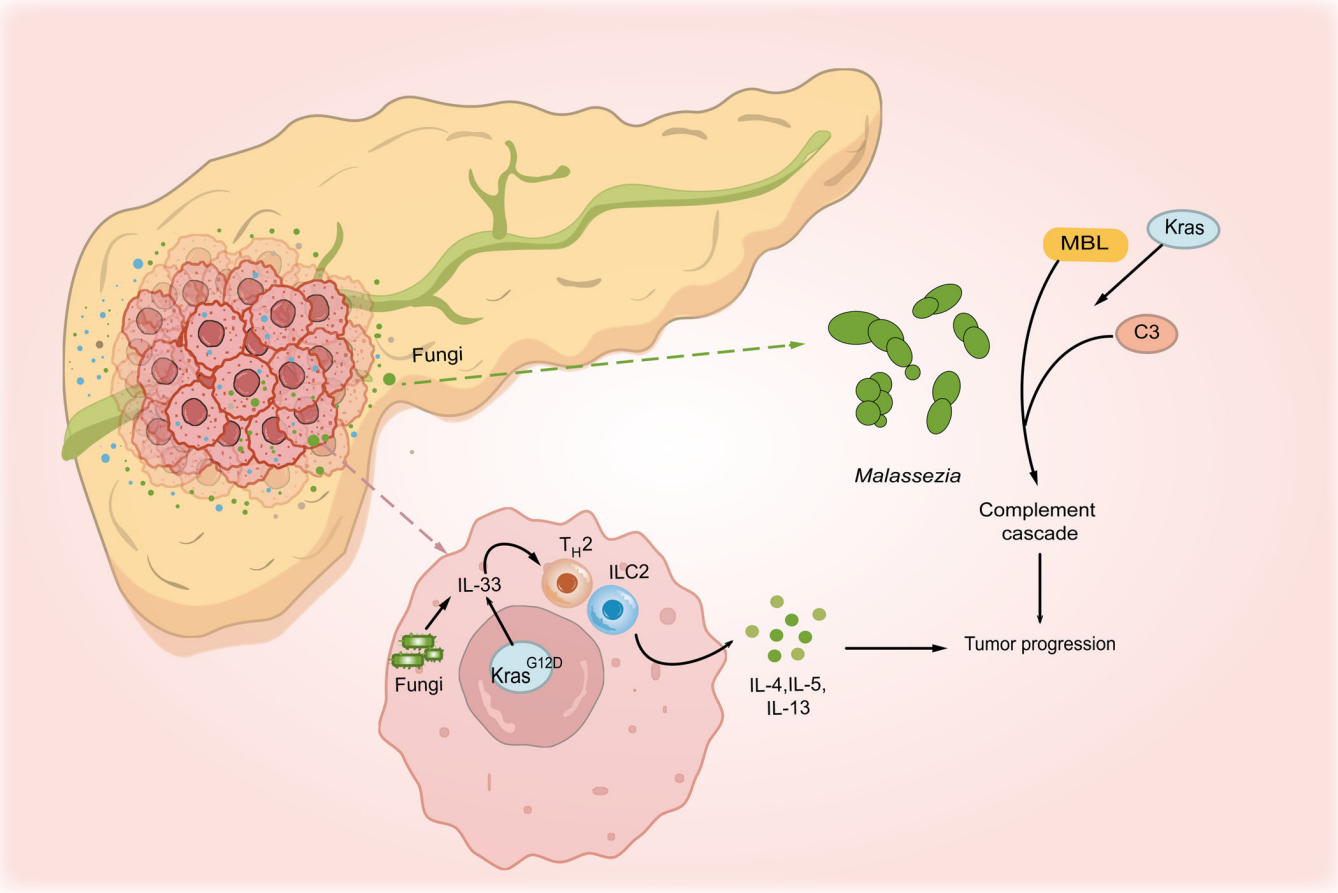
Graphical illustration of multiple processes by which fungi participate in the progression of pancreatic ductal adenocarcinoma cancer. *Malassezia* can promote carcinogenesis by stimulating the activation of MBL to drive the complement cascade, and oncogenic *Kras* could activate the MBL‐C3 cascade to enhance the proliferation of the tumor. Intratumoral fungi like *Alternaria alternata* can promote IL‐33 secretion from PDA cells. Oncogenic *Kras* ^G12D^ also elevates IL‐33 expression, and then recruits and activates T_H_2 and ILC2 cells. This finally promotes the secretion of protumorigenic cytokines, such as IL‐4, IL‐5, and IL‐13, finally provoking tumor progression. IL, interleukin; ILC2, lymphoid cells 2; MBL, mannose‐binding lectin; PDA, pancreatic ductal adenocarcinoma.

Notably, oncogenic *Kras* ^G12D^ also elevates IL‐33 expression in PDA cells, thereby recruiting and activating T_H_2 and lymphoid cells 2, which in turn can provoke tumor growth through the secretion of protumorigenic cytokines, such as IL‐4, IL‐5, and IL‐13 [10]. Surprisingly, intratumoral fungi like *Alternaria alternata* can promote IL‐33 secretion from PDA cells, and both *M. globosa* and *A. alternata* promote tumor growth simultaneously [10] (Figure 4).

#### Gastric cancer (GC)

GC stands as the fourth most common malignant tumor in the world and remains a leading causes of cancer‐related deaths [36, 37]. It has been shown that several host‐related factors, including age, cigarette smoking, genetic susceptibility, environmental factors, and microbial infections, are associated with the development of GC [38]. However, due to the challenge of culturing the commensal microbiota residing in the stomach [39], the relationship between gastric microbiota (such as fungus) besides *Helicobacter pylori* and GC has not been thoroughly investigated.

GC is associated with gastric fungal dysbiosis characterized by changes in ecology and composition. One fungus that can be used as a biomarker for GC is *C. albicans* because it was found to be greatly enhanced in GC. In addition to *C. albicans*, *Fusicolla acetilerea*, *Arcopilus aureus*, and *Fusicolla aquaeductuum* rose in abundance, while *Candida glabrata*, *Aspergillus montevidensis*, *Saitozyma podzolica*, and *Penicillium arenicola* had significantly lower abundance [40]. Furthermore, by decreasing the abundance and diversity of fungus in the stomach, *C. albicans* could play a role in the development of GC [40]. Increased *Candida* has also been found to be associated with an enrichment of genes involved in cytokine interactions, host immunity, and inflammation, including IL1A, IL1B, IL6, IL8, CXCL1, CXCL2, and IL17C [6]. This proinflammatory immunological profile aligns with previous research demonstrating that *C. albicans* induces infiltration of IL‐1β, neutrophils, and Th17 cells in the gut [41]. Additionally, a high prevalence of *Candida* DNA was strongly associated with reduced survival, implying that *Candida* DNA may serve as a prognostic biomarker for gastrointestinal tumors [6]. These results highlight the significance of stomach fungal homeostasis. Further investigation is warranted to elucidate the prospective contribution of fungi, including *C. albicans*, in gastric carcinogenesis and to determine their utility as noninvasive biomarkers for diagnosing GC.

#### Liver cancer

Liver cancer, particularly hepatocellular carcinoma (HCC), has become the third leading cause of cancer‐related deaths [1]. Compared with the healthy controls, dysbiosis of gut mycobiota were discovered in HCC patients, characterized by reduced alpha diversity and increased abundance of opportunistic fungi, such as *Malassezia* and *Candida*. The abundance of *C. albicans* was also significantly higher in tumor node metastasis stage III‐IV HCC patients compared with stages I–II, while the symbiotic fungus *S. cerevisiae* showed the opposite trend. Besides, abnormal colonization by these opportunistic pathogenic fungi could lead to metabolic changes and accelerated progression of HCC [42, 43].

In addition, several fungi, including *A. flavus* and *Aspergillus parasiticus*, can produce more than 20 different types of aflatoxins. These toxins are widely present in various crops, such as cereals, oilseeds, nuts, and spices, with the liver being their primary target organ [44, 45]. The International Agency for Research on Cancer has identified aflatoxins as carcinogenic (Group 1) and potentially carcinogenic (Group 2B) to humans [46]. Short‐term exposure to high concentrations of aflatoxins can lead to acute hepatitis and even death, while chronic exposure could cause immunosuppression, trophic disturbance, and tumor [47]. Aflatoxin B1 (AFB1) is the most harmful of these toxins and it has been linked to 4.6%–28.2% of HCC cases worldwide [48]. AFB1 epoxidation is known to be a crucial part of the genotoxic process and, consequently, in the progression of cancer. It is primarily metabolized in the liver by CYP450 enzymes and converted to reactive 8,9‐epoxide. This metabolite has a strong affinity for DNA, leading to the formation of adducts that promote DNA mutations and contribute to the development of HCC [44, 49], with the main mutation being a G→T reversal at the original adduct site [50]. Additionally, aflatoxin may also synergize with other risk factors, such as hepatitis B infection, further increasing the risk of HCC [51].

#### Oral cancer

The oral cavity harbors the second largest microbiota in the human body, known as the oral microbiota [52], where *Candida* is the most prevalent fungi, followed by *Aspergillus*, *Fusarium*, and *Cryptococcus* [53]. Of note, a dysbiotic mycobiota was found in association with oral squamous‐cell carcinoma, accompanied by an expansion of *Candida* species of which *C. albicans* was the most common [54].

Additionally, it has been demonstrated that *C. albicans* isolated from the oral cavity can produce toxic and carcinogenic acetaldehyde from ethanol, a Group 1 carcinogen that can cause mutations by directly binding to DNA [55, 56]. Acetaldehyde‐producing *Candida* from high ethanol sources is more common in oral cancer patients, and this upregulation of acetaldehyde metabolism is associated with smoking and alcohol consumption. In addition, *Candida* can form biofilms and produce hydrolytic enzymes, all of which are risk factors for oral cancer [57, 58].

Certain conditions such as a depressed immune status may lead to the formation of nitrosamines from the precursors of various *C. albicans* that are capable of nitrosation. These nitrosamines then act on the normal epithelium, resulting in dysplasia and ultimately carcinogenesis. Additionally, several proto‐oncogenes may be activated by nitrosamines alone or in conjunction with other chemical carcinogens, thereby initiating the progression of malignant lesions. While smoking promotes keratinization of the oral mucosa further enhancing *C. albicans* colonization, tobacco products can also lead to epithelial changes through nitrosation. Thus, the combination of *C. albicans* and tobacco may enhance the carcinogenic process [59]. However, the mechanism of carcinogenesis by *Candida*‐produced nitrosamines is not sufficiently precise, and more extensive studies in different cancer models and environments are needed in the future.

### Nondigestive system cancers

#### Lung cancer

**Figure 5:**
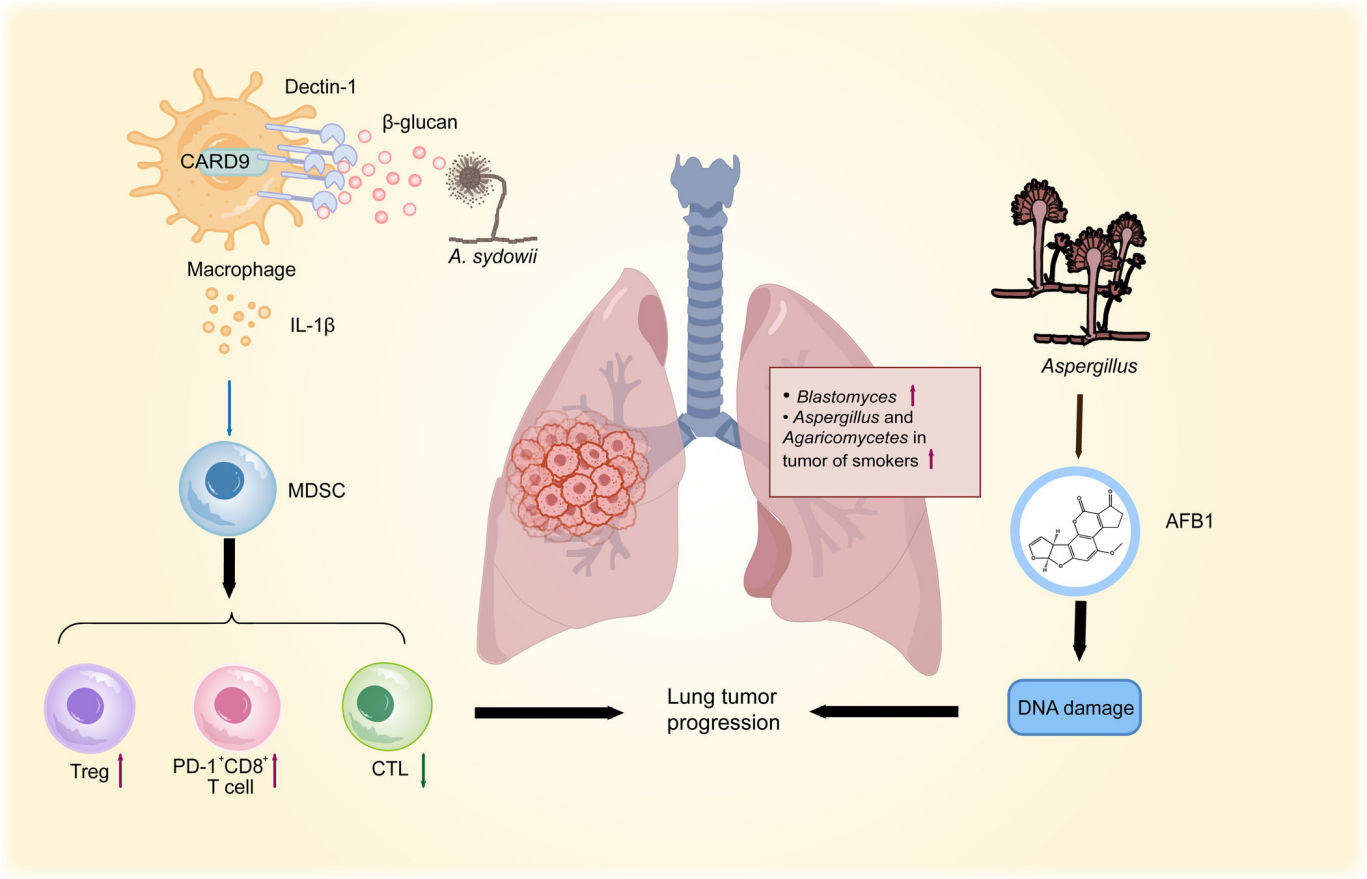
Graphical illustration of the functional role of fungi in the development of lung cancer. *Blastomyces, spergillus*, and *Agaricomycetes* were enriched in tumors. Intratumor mycobiome, especially *Aspergillus sydowii*, could boost the recruitment and activation of MDSCs which was mediated by IL‐1β secretion via β‐glucan/Dectin‐1/CARD9 pathway. This causes suppression of CTL cells activity and aggregation of Tregs and PD‐1^+^ CD8^+^ T cells, finally resulting in lung cancer progression. AFB1 is able to cause significant oxidative DNA damage as well as stimulate the migration of lung cancer cells. AFB1, Aflatoxin B1; CARD9, caspase‐recruitment domain 9; CTL, cytotoxic T lymphocyte; IL, interleukin; MDSCs, myeloid‐derived suppressor cells; Tregs, T regulatory cells; ↓, decrease; ↑, increase.

Lung cancer remains the top cause of cancer deaths worldwide [1]. In addition to hereditary factors and environmental factors like smoking and air pollution associated with the development of lung cancer [60, 61], recent studies have discovered a relationship between the enrichment of specific species of fungi in lung tumors. The Cancer Genome Atlas cohort found *Blastomyces dermitidis*/*gilchristii* was enriched in cancer patients, and notably, a higher abundance of *Aspergillus* and *Agaricomycetes* was found in tumors of smokers in the Weizmann cohort compared with lung cancer patients who had never smoked [6, 8]. Notably, Liu et al. have recently identified the importance of intratumor mycobiota, especially *Aspergillus sydowii*, in facilitating the progression of lung adenocarcinoma. *A. sydowii* boosts the recruitment and activation of MDSCs which were mediated by IL‐1β secretion via β‐glucan/Dectin‐1/CARD9 pathway. This causes suppression of cytotoxic T lymphocyte cells activities and aggregation of T regulatory cells and PD‐1^+^ CD8^+^ T cells subsequently, resulting in cancer development eventually [12]. Even so, the temporal sequence between *A. sydowii* colonization and carcinogenesis is poorly understood, and it is still unclear how the pathogen enters human lung cancers. Furthermore, it will also be crucial to validate these findings in more extensive and diverse cohorts, and translate them into clinical treatments [62] (Figure 5).

Moreover, AFB1 is a fungus metabolite that can cause cancer when it is inhaled into the lungs through grain dust, hence it is also regarded as a lung cancer carcinogen [63]. In different experimental models, researchers recently have discovered that AFB1 induces the production of DNA‐binding metabolites in lung cytosols that can increase lung susceptibility to AFB1 [64]. AFB1 is also able to cause significant oxidative DNA damage that is associated with the carcinogenicity of AFB1 to the lung, as well as stimulate the migration of lung cancer cells [65, 66]. In addition, cytochrome CYP2A13 was engaged in metabolizing AFB1 to its carcinogenic AFB1‐8,9‐epoxide, which is a key player in inhalation‐related lung cancer [67]. Overall, these findings showed that research has been done on the relationship between lung cancer and AFB1, but the potential significance of Aflatoxin M1 needs to be explored further.

#### Breast cancer

Female breast cancer is the most common cancer, and one of the top five cancers with the highest mortality rate [1]. It has previously been confirmed that fungus and breast cancer are related. Compared with other tumors, *Malassezia* was significantly enriched in breast cancer and there was a fungi–bacteria co‐occurrence with *Aspergillus* and *Malassezia* as the hub. In addition, an elevated prevalence of *M. globosa* in tumors was associated with reduced overall survival [6, 8]. It is also interesting to note that each subtype of breast cancer had relatively distinct fungal characteristics. Breast cancers can be categorized according to their surface receptors as endocrine receptor (ER; estrogen or progesterone receptor) positive (BRER), human epidermal growth factor receptor 2 (HER2) positive, triple‐positive (BRTP: estrogen, progesterone, and HER2 receptor negative), and triple‐negative (BRTN) [68]. Banerjee et al. found that only seven fungal families (*Aspergillus*, *Candida*, *Coccidioides*, *Cunninghamella*, *Geotrichum*, *Pleistophora*, and *Rhodotorula*) were shown to be present in more than one breast cancer subtype. Compared with the BRTN samples, the receptor‐positive cancer samples (BRER and BRTP) revealed more intricate fungal diversity [68]. The authors' subsequent findings were consistent with previous ones in that the BRER samples showed the largest fungal diversity, among which *Arthroderma* were accountable for the highest average hybridization signal. Whereas the BRTN samples had the lowest diverse fungi and yeasts along with skin fungi made up most of the fungal signals [69]. In a mouse model of breast cancer, an important study demonstrated the impact of fungi on cancer treatment. It was found that intestinal fungi can modulate the antitumor immune responses following radiation therapy. The administration of antifungal antibiotics like fluconazole or 5‐fluorocytosine showed that antifungal treatment, as opposed to antibiotic treatment, enhanced the efficacy of radiation therapy. This was evidenced by delayed mammary tumor growth and increased survival rate in the mice model [70]. The current insufficient research on the contribution and evaluation of fungi to cancer treatment suggested novelty perspectives for future exploration.

The investigation of cancer‐related fungi is still in its early stages, and the field of cancer fungal biomes is rapidly expanding. However, the heterogeneity in research methods and sample populations has imposed certain limitations on these studies. One crucial aspect that requires further investigation is the causal relationship between cancer‐associated fungal features and the development of cancer, as it remains unclear whether these features are a cause or a consequence of cancer processes. Future research in this area holds great promise and will shed more light on the complex interactions between fungi and cancer.

## NUTRITIONAL REGULATION OF MYCOBIOME AND CANCER PROGRESSION

Accumulating evidence has suggested that commensal and pathogenic fungi possibly involved in regulation of oncogenesis progress, through secreting bioactive molecules, cross‐talking with nearby bacteria and modulating host immunity. It is recognized that all lifestyles, environmental and genetic variables have a significant impact on mycobiome [71, 72, 73]. Diets significantly influence the intestinal metabolic environment, altering the lifestyle of fungi. Few intratumoral fungi could be affected by the abnormal microenvironment after tumor metabolic reprogramming. In light of diet, mycobiome likely represents a putative correlation between nutrients and cancer, though the data are inconclusive yet (Table 1). Dietary nutrients (i.e., cholesterol, carbohydrates, proteins, and minerals), play crucial physiological roles in growth, reproduction, and invasive pathogenesis of the fungal microbiome: (i) Fungus could sense nutritional alterations specifically in microenvironment and change adaptively. (ii) Consequently, fungi produce distinct metabolic products in response, which may serve as signaling molecules, interacting with bacterial kingdom and neighboring cells. (iii) Fungi also adapt their lifestyle, as seen in transforming from commensal to pathogen. (iv) Fungal resistance against antifungal agents may be manipulated by nutrients. It is warranted to explore the effects of nutrients on fungi and oncogenesis. The key aspects of the research involve: first, uncovering the receptors through which fungi sense nutrients and the signaling pathways they utilize to alter their lifestyle. Second, exploring how fungi metabolize nutrients, secrete secondary metabolites, and influence the surrounding environment.

**Table 1:** Dietary nutrients modulating fungal composition, colonization and cancer risk.

Diet	Fungal abundance alteration	Fungal phenotype conversion	Cancer risk
Alcohol intake	*Candida*↑	The pathogenicity of *Candida* for ALD↑	Liver cancer risk↑
	*Epicoccum*, *Galactomyces*, and *Debaryomyces*↓		Pan‐cancer risk↑
Fat and cholesterol	*Saccharomyces cerevisiae*↓	17‐β‐estradiol can lead to Candidiasis	Liver cancer risk↑
	*Basidiomycota*↑		
Sugar and starch	*Methanobrevibacter, Candida*↑	Different glycogen led to different phases of transformation	Cancer risk↑
Fiber	*Prevotella, Candida*↑	–	Cancer risk↓
Protein	*Methanobrevibacter, Candida*↓	Nitrogen source can affect fungal phase, biofilm formation, and antifungal drug tolerance	Cancer mortality↑
Minerals	–	“Nutrition tug of war”	–

Abbreviations: ALD, alcohol‐related liver diseases; ↑, increase; ↓, decrease.

### Alcohol intake

Around the world, alcoholic beverages are special dietary components, often consumed with food. Alcohol contains seven calories for each gram consumed and can be considered as an obesogenic factor. Epidemiological evidence has proved that alcohol consumption contributes to about 4% of cancer cases globally. Drinking alcohol increases the risk of several types of cancer, including CRC, liver cancer, breast cancer, and so forth [74]. The composition and interaction of bacterial and fungal species could be altered owing to chronic alcohol intake [75]. Ethanol produced by *C. albicans* induces the production of 5‐methyl‐phenazine‐1‐carboxylic acid in *Pseudomonas aeruginosa*, which exhibits antifungal activity [76]. Previous studies investigated that chronic ethanol intake significantly altered mycobiome community, with a significant overgrowth of *Candida* [75, 77]. The colonization of *C. albicans* is associated with various intestinal diseases, such as inflammatory bowel disease [78, 79]. Alcohol increases intestinal *C. albicans*‐specific Th17 cells, migrating to the liver and promoting liver disease via IL‐17 receptor a signaling in Kupffer cells [80]. Also, chronic alcohol administration generates fungal dysbiosis and *Candida* overgrowth, leading to fungal β‐glucan translocation. Translocation of β‐glucan induces IL‐1β secretion via the C‐type lectin‐like receptor CLEC7A on Kupffer cells, subsequently contributing to hepatocyte damage and promoting the development of ethanol‐induced liver disease [75]. Likewise, it has been demonstrated that the intestinal microbiota produces endogenous ethanol which is obscured by the first‐pass effect, considered as an etiology of nonalcoholic fatty liver disease [81]. Ethanol and its metabolite acetaldehyde, give rise to alcohol‐mediated carcinogenesis via inflammation, genetic damage and metabolism impairment. Also, *C. albicans* leads to alcoholic‐associated liver diseases via upgraded specific Th17 cells in response to alcohol [80]. Other putative pathogenesis pathways of fungi in liver diseases may depend on or secreting candidalysin [82, 83] and inducing prostaglandin E2 [84]. In spite of fungal etiology mediated by alcohol in liver disease, the causality in tumorigenesis of primary liver cancer remains unclear, and more insightful research would be of great assistance.

### Fat and cholesterol

Dietary cholesterol and fat produce more calories per gram which double the amount of carbohydrates. Epidemiology statistics and preclinical studies both have reported a significant positive correlation between fat and cholesterol intake with the risk of liver cancer [85, 86, 87]. It has been reported that the occurrence of obesity is associated with gut fungi. *Candida* parapsilosis promotes the accumulation of fat and the development of obesity by producing lipase and converting dietary triglycerides into monoacylglycerols [88]. However, whether fungi, like bacteria, can contribute to carcinogenesis independently of obesity remains unclear. Also dietary fat is potential hazard for metabolic syndromes by altering the microbiome and mycobiome make‐up. It has been revealed that a high‐fat diet could lead to a shift in mycobiota composition in the mice model (i.e., the abundance of *S. cerevisiae* declined) [14]. Specific cholesterol, such as estrogen, can serve as signaling molecules inducing distinctive phenotypic changes in fungi. Phytoestrogen, an estrogen similarity from legumes, grains, and several veggies, can interfere with steroid biosynthesis [89, 90, 91]. For instance, it has been reported that 17‐β‐estradiol can stimulate the growth and colonization of *C. albicans*, leading to Candidiasis [92, 93, 94]. Conversely, 17‐β‐estradiol inhibits the development and delays the morphogenesis *Paracoccidioides* [95]. Thus, dietary fat, cholesterol, and steroid hormone levels may lay a profound effect on mycobiota composition.

### Sugar and starch

Sugar and starch (a polysaccharide comprising glucose monomers) are two major types of dietary carbohydrates (DCHO), which were recommended to make up 45%–65% of total daily calories. Overconsumption of sugar fuels the growth of cancer indirectly via excessive caloric intake. Low‐carbohydrates diets were confirmed to modulate the microbiome composition accompanying weight loss [96], whereas carbohydrates restriction may have a potential benefit for tumor prevention. The typical low‐carbohydrate diet can induce weight loss, intensively associated with gut bacterial and fungal covariation [96]. A previous study emphasized that *Methanobrevibacter* and *Candida* abundance were positively associated with recent high‐carbohydrates diets, despite bacterial population structure was predominantly with long‐term one [97]. Experiments revealed that different glycogen affects the substrate composition and hypha transformation of *C. albicans* in vitro [98]. *C. albicans* sense external glucose concentration changes and activate MAPK or cAMP signaling pathways to promote mycelium formation and induce phenotypic switching [15, 99]. Glucose, fructose, maltose, sucrose, and galactose were able to induce the conversion of *C. albicans* from the yeast phase to the mycelial phase, whereas lactose, sorbitol, and glycerol were unable [15]. In the mice model, different carbon sources led to differences in mycotoxicity [100]. Overall, mice fed with sugars such as glucose or galactose resulted in less severe fungal infections, while higher mycobacterial loads presented in mice with lactic acid [101]. This holds significance in the context of acute fungal infections: restricting the intake of fructose and sucrose over the short‐term aids in controlling fungal growth. Additionally, given the role of fungi in tumor development, carbohydrate restriction and calorie limitation have a potential role in slowing tumor progression.

### Fiber

Fiber also is a complex carbohydrate. In contrast to the “notorious” sugar, dietary fiber consumption was putatively suggested to protect against obesity and help lower cancer risk [102]. Short‐chain fatty acids (SCFAs), fermented from dietary soluble fibers by gut microbiota, can directly activate G‐protein‐coupled receptors, inhibit histone deacetylases, and serve as energy substrates to connect dietary patterns and gut microbiota, thereby improving the intestinal health and inhibiting intestinal cancer development [103]. It was observed that *Saccharomycetales* spp. had a positive association with bacterial SCFA producers (i.e., *Clostridium*, *Faecalitalea*, and *Megamonas*). Consistently, *Saccharomycetales* spp. was also positively associated with acetic acid and butyric acid. Nevertheless, more mechanistic work is needed to understand the relationship among gut *Saccharomycetales* spp., bacteria, SCFAs and health [104].

### Protein and nitrogen sources

High‐protein intake was associated with a fourfold increased risk of cancer mortality in people aged 50–65 [105]. However, meta‐analyses have not found evidence of a detrimental effect of high‐protein intake on prostate, ovarian, and colorectal [106, 107, 108]. In spite of that, a high‐protein diet is considered as an essential nutritional support for adjunctive treatment of infectious diseases and cancer. *Methanobrevibacter* and *Candida* abundance were negatively associated with high‐protein diet, contrary to the effects of DCHO [97]. Nitrogen anabolism or catabolism is an essential metabolism pathway, consistently plays an important role in protein synthesis of both human and microbiota, including *C. albicans* and *Aspergillus fumigatus*. Current studies have shown that nitrogen source can affect the phase transition and biofilm formation of *C. albican*s [109, 110, 111, 112]. *C. albicans* utilize the Csh3‐SPS sensing complex (Csh3‐Ptr3‐Ssy5 sensing pathway) to perceive extracellular amino acid levels and regulate the secretion of virulence factors via Stp1 controlled genes, including the major secreted aspartyl protease SAP2 [113]. *C. albicans* can also facilitate proline metabolism via expressing proline metabolic enzymes to promote the transition from the yeast phase to the hyphal phase when engulfed by macrophages. Inhibition of proline metabolism affects hyphal formation, weakening *C. albicans*' ability to escape macrophages [112]. Similarly, CpcA, a functional gene in *A. fumigatus*, is capable of modulating the expression of virulence factors in response to amino acid stress conditions [114].

### Minerals

It is reported that deficiencies of necessary vitamins and minerals increase susceptibility to several types of cancer. Whereas high dose vitamin or mineral supplements have not reduced cancer risk in well‐nourished populations and might increase risk [17]. In view of the rarity and necessity of minerals, especially micronutrients, for both host and fungi, the “nutrition tug of war” between them is more intense than macronutrients. Vertebrate hosts have evolved complex metal restriction strategies to prevent fungal protein synthesis and disrupt cellular homeostasis, a process termed “nutritional immunity.” In turn, fungal pathogens have developed adaptive mechanisms to cope with conditions of metal depletion or excess [115, 116]. Minerals, including potassium, calcium, sodium, magnesium, iron, copper, and zinc, play a crucial role in protein structure, enzyme catalyst reactions, signal transduction pathways, and cellular physiological homeostasis in both host and fungi [117, 118, 119, 120, 121]. Moreover, it is first discovered that phosphorus nutrition is another nutrient in the regulation of the target of rapamycin (TOR) signaling pathway [122]. *C. albicans* sense oxidative‐stress pressure in different microenvironments, exert virulence to invade the host, and resist antifungal drugs through the phosphate transporter receptor Pho84 and the downstream TOR signaling pathway. In particular, homologous protein of Pho84 were not observed in humans, but the Pho84 gene sequence is highly conserved in the fungi [123, 124].

## FUNGUS TARGETED PRECISION NUTRITIONAL THERAPIES AGAINST CANCER

Dietary factors, including obesity, alcohol consumption, insufficient fiber, and processed meat, are ranked among the top 10 attributing factors of cancer. In this case, dietary modifications and interventions have gained significant attention in the realm of cancer prevention and treatment. Fasting has been observed to have beneficial effects on tumor incidence, cancer progression, and the reduction of chemotherapy‐related adverse effects [125, 126]. Similarly, prolonged caloric restriction holds promise as a feasible approach with similar advantages [127, 128]. Carbohydrate Restriction (or Ketogenic Diet), Mediterranean diet (containing high concentrations of monounsaturated fatty acids and other potentially beneficial components), Protein restriction, and vegetarianism may provide beneficial effects on cancer risk and outcomes, although further research is needed to draw definitive conclusions [129, 130, 131, 132]. Despite the limited data, dietary interventions can slow the progression of cancer, improve the efficacy of chemotherapy and reduce the risk of later complications. While general dietary support has shown promise, the lack of precision remains a limiting factor. An in‐depth understanding of the interactions between nutrients and fungal pathogenesis will provide better guidance for clinical application.

### Reducing the virulence of pathogenic fungi via targeting nutritional sensor‐effector pathway

Among the most well‐known pathogenic fungi, *Candida*, *Malassezia*, and *Aspergillus* species cannot be neglected in tumorigenesis and cancer metastasis. *C. albicans*, as previously mentioned, have been implicated in promoting cancer progression in oral, gastrointestinal, and CRCs [24, 54]. *Malassezia* has been associated with poor prognostic outcomes in breast cancer [8], while *A. sydowii* has been recently linked to disease progression and nonsurviving in patients with lung cancer [12]. In spite of antifungal resistance in the treatment of cancer, hepatic toxicity and drug resistance are two other major challenges. Consequently, the antagonism of fungi through dietary interventions has great potential for tumor control.

First, quorum sensing (QS), a density‐dependent signaling mechanism of microbial cells, governs a variety of essential processes, including pathogenicity, morphogenesis, and filamentation [133]. The discovery of QS in fungi, exemplified by the control of filamentation in the pathogenic polymorphic fungus *C. albicans* through farnesol, highlights the significance of QS as a potential therapeutic target [134, 135]. Since QS is indirectly involved in the emergence of multiple drug resistance in microbial pathogens, it is necessary to find alternative antimicrobial therapies that target and inhibit QS.

**Figure 6:**
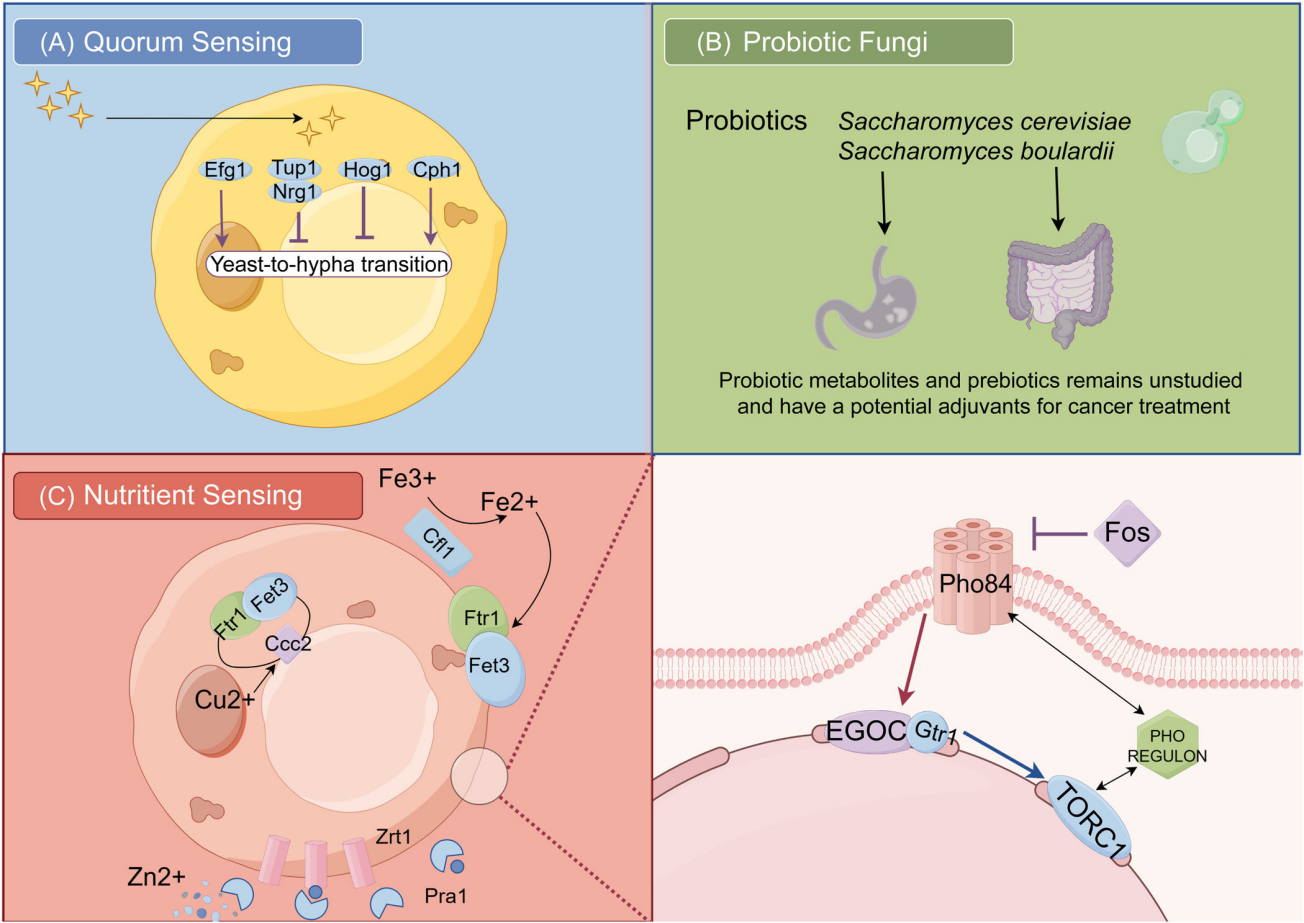
Potential therapeutic insights into tumor prevention and precision nutritional therapy. (A) Quorum sensing (QS) could play an essential role in dietary intervention against fungal virulence. (B) Blockade of specific metabolic pathways would be an effective way to fight against fungi with minimal side effects. (C) It holds great promise for anticancer therapy by supplementing beneficial metabolites and probiotics, such as *Saccharomyces cerevisiae* and *Saccharomyces boulardii*. EGOC, the EGO complex; Fos, foscarnet; PHO REGULON, a huge regulatory network in bacteria; TORC, target of rapamycin complex 1.

Second, metal receptors have emerged as promising drug targets for novel therapeutics because of the significance of metal uptake mechanisms for microbiota growth in the host environment. Pho84, for instance, can be a specific antifungal drug target. Inhibiting the expression of Pho84 not only demonstrates effective antifungal effects, but also simultaneously enhances the efficacy of antifungal drugs and reduces the virulence of *C. albicans* [122, 123, 124] (Figure 6).

### Developing new antitumor therapies through fungal secondary metabolites and prebiotics

Advances in genomics, metabolism and microbiome are improving our understanding of cancer metabolic diversity, leading to noval cancer therapies based on precision nutritional therapy (PNT). With regard to bacteria, adjuvants for cancer treatment such as facal microbiota transplantation, prebiotics, engineered probiotics, and metabolites have been applied in bench [53]. Similarly, it holds great promise for anticancer therapy by supplementing probiotic fungi and downstream metabolites, as well as combating fungi through specific blockades of nutrient metabolism. The exploration of the relationship between nutrients and pathogen–host interaction mechanisms of fungi can provide new ideas for the treatment of cancer.

In addition to pathogenic fungi, certain beneficial fungi like *Saccharomyces boulardii* have shown potential in attenuating liver injury and protecting against diseases [136, 137]. Nonetheless, it is essential to address side effects associated with their clinical use, particularly in vulnerable populations [138]. Exploring probiotics with precision offers the possibility of individualized treatment and more refined approaches to PNT.

Furthermore, fungal secondary metabolites have always held great promise. The study of fungal secondary metabolites, driven by advances in molecular biology, genomics, and bioinformatics, has entered a new era. Fungi produce secondary metabolites in response to variable environmental stresses, and thus secondary metabolite production is controlled by a complex regulatory network [139].

Recently, metabolism enzymes or bioactive compounds have been synthesized using modified microbes. Researchers employed chassis to express functional genes or gene clusters in a heterogeneous manner to address cancer. To provide more accurate and effective treatment results, scientists produced “smart” bacteria that can detect and react to microenvironmental signals [140, 141]. The application of live mycobiome and engineered strains implies that retaining sensor–receptors and immunogenicity is more conducive to the targeted therapies involving fungal secondary metabolites and prebiotics.

## CONCLUSION AND FUTURE PERSPECTIVES

An expanding understanding of the connection between the human mycobiome and cancer revealed potential mechanisms and avenues for treatment. Fungi have been found to interact with nutrients, contributing to a complex network that significantly influences host physiology. However, it is important to remember that these aspects are still in their early stages of research and exploration.

To date, investigations have revealed superior predictive accuracy and sensitivity based on bacteria and fungi in gauging cancer prognosis, although the fungal microbiota has received less attention due to its lower diversity and abundance relative to bacteria. The potential for utilizing fungi and nutrients as biomarkers or therapeutic targets in modulating cancer development, progression, and treatment response holds immense promise. Nonetheless, several key questions remain unanswered. First, it is crucial to determine whether the association between fungi and cancer‐related processes is causal or consequential. This would provide valuable insights into the underlying mechanisms. Furthermore, the role of nutrient‐mediated machinery in the fungal flora's influence on carcinogenesis, metastasis, therapeutic effects and drug resistance remains largely unexplored. In this pursuit, more extensive and rigorous validation tests encompassing mycobiome composition and fungal‐bacterial interactions are warranted, necessitating larger‐scale sample sizes and multicenter cohort studies. Additionally, the development and integration of novel technologies and the application of multiomics approaches, such as high‐resolution deep mycobiome sequencing and the combination of single‐cell sequencing with spatial transcriptomics, are imperative for advancing our understanding.

Effective metabolic adaptation to the host microenvironment is crucial for fungal proliferation during infections. As the understanding of the interactions between nutrients and the expression of specific genes deepens, novel cancer therapies rooted in precision nutrition strategies may emerge. Metal receptors and QS molecules have become promising drug targets for the development of innovative therapeutics. Designing dietary regimens tailored for precision therapy against fungal targets has great value for clinical application.

Hence, delving into the intricate relationship between nutrients and the pathogenic mechanisms of fungi can provide novel insights into the mechanistic exploration and treatment of cancer. Although we find ourselves at the dawn of a new era, the research will serve as a foundation for translating individual treatment and PNT from the realm of theory to practical implementation.

## AUTHOR CONTRIBUTIONS

Di Wu and Yun‐Xuan Guan wrote the manuscript and drafted the figures. Chen‐Hao Li wrote the manuscript. Quan Zheng revised the manuscript. Zuo‐Jing Yin revised the manuscript. Hui Wang supervised and funded this project. Ning‐Ning Liu wrote, supervised and funded this project. All authors have read the final manuscript and approved it for publication.

## CONFLICT OF INTEREST STATEMENT

The authors declare no conflict of interest.

## Data Availability

This paper does not generate any new data. Supporting information materials (graphical abstract, slides, videos, Chinese translated version and update materials) may be found in the online DOI or iMeta Science http://www.imeta.science/.
